# A neurological perspective on Tarsila do Amaral's “Abaporu”: migraine aura inspiring art?

**DOI:** 10.1055/s-0045-1814376

**Published:** 2026-01-25

**Authors:** Wallyson Pablo de Oliveira Souza, Patrick Emanuell Mesquita Sousa-Santos, Juliane Prieto Peres Mercante, Mario Fernando Prieto Peres, Raimundo Pereira Silva-Néto

**Affiliations:** 1 Universidade Federal do Delta do Parnaíba, Parnaíba PI, Brazil.; 2 Universidade Estadual Paulista “Júlio de Mesquita Filho”, Faculdade de Medicina de Botucatu, Botucatu SP, Brazil.; 3 Universidade de São Paulo, Faculdade de Medicina, Hospital das Clínicas, São Paulo SP, Brazil.; 4 Universidade de São Paulo, Instituto de Psiquiatria, São Paulo SP, Brazil.

**Keywords:** Migraine with Aura, Migraine Disorders, Art, Medicine in the Arts

## Abstract

**Background:**

Tarsila do Amaral's painting “Abaporu” is a seminal work in Brazilian modernist art, yet its interpretation continues to intrigue scholars due to its complex symbolism.

**Objective:**

To examine “Abaporu” through the perspective of neuroesthetics, exploring potential parallels between its visual elements and sensory disturbances characteristics of migraine aura.

**Methods:**

A structured review of primary and secondary art-historical sources and a systematic literature search in PubMed and Google Scholar were conducted. The analysis focused on Amaral's recurrent use of disproportion and gigantism in the 1920s and on evaluating the plausibility of a neuroesthetic hypothesis in this art-historical context.

**Results:**

Our analysis indicated that the gigantism and deliberate deformation in “Abaporu” belong to a consistent program already evident in Amaral's 1920s painting. From this perspective, the painting's oversized feet and hands contrasting with a diminutive head evoke perceptual alterations reminiscent of macropsia and micropsia, phenomena linked to Alice in Wonderland Syndrome and migraine aura. Its vibrant palette and melancholic undertones, likewise, resound the sensory disturbances and affective dimensions associated with migraine, suggesting that Amaral's programmatic distortions also invite a neuroesthetic reading.

**Conclusion:**

While no evidence supports a medical diagnosis of migraine in Amaral, the visual motifs and techniques in “Abaporu” can be read both as deliberate esthetic strategies of her Pau-Brasil and Anthropophagic phases and as intuitively resonant with neurological models of altered perception. This dual lens enriches our understanding of art's capacity to embody complex perceptual experiences and encourages further interdisciplinary dialogue between art history and neuroscience.

## INTRODUCTION

Tarsila do Amaral (1886–1973), revered as Brazil's most preeminent painter, left an indelible mark on modern art with her innovative approach and profound cultural influence. Among her notable works, “Abaporu,” painted in 1928, stands as a seminal piece that embodies the essence of Brazilian modernism. Depicting a monumental figure with oversized hands and feet juxtaposed against a diminutive head, the painting is often interpreted as symbolizing the primacy of physical labor over intellectual pursuits. 1 2 However, this interpretation invites a deeper exploration from a neurological standpoint.

According to the *International Classification of Headache Disorders, Third Edition* (ICHD-3), typical migraine aura comprises transient, fully reversible neurological symptoms, such as visual, sensory, or language changes, which precedes or occurs during headache, appear gradually, spread, and then disappear, lasting from 5 minutes to 1 hour. 3 Visual aura is the most common phenotype of aura in migraine aura, occurring in more than 90% of the cases. 4 It often presents as a fortification spectrum, flickering scotomas, hemianopsia, foggy/blurred vision, flashing lights, dots, wavy lines, blind spots, and tunnel vision. These symptoms can be noticed throughout the visual field or in just limited regions. 5 6 7 8 9

Interestingly, these perceptual anomalies bear a striking resemblance to the symptoms described in Alice in Wonderland Syndrome (AIWS), an entity initially described in 1955 by psychiatrist John Toddy (1914–1987) as a perception disorder characterized by visual distortions, when individuals unrealistically perceive the size of some parts of their bodies and other objects. The syndromic nomenclature alludes to Lewis Carroll's novel *Alice's Adventures in Wonderland* , in which the protagonist notices, among other things, changes in the shape of her body, sometimes getting bigger, sometimes smaller. 10 Such changes in perception are known, respectively, as macropsies and micropsies, and are commonly associated with migraine patients, especially those who present with migraine aura. 11 12 Some studies suggest that micropsia is present up to 70% of the time, while macropsia may occur in about 38% of cases in patients experiencing migraine aura and AIWS. In childhood, AIWS has often been linked to encephalitis resulting from the Epstein-Barr virus and migraine. In adulthood, migraine is frequently considered the main underlying factor. It is suspected that the protagonist is an alter-ego of Carroll, who perhaps suffered from the syndrome himself. 13 14

In the course of art history, several other artists, including Giorgio De Chirico (1888–1978), Kaethe Kollwitz (1867–1945), Sarah Raphael (1960–2001), and possibly Pablo Picasso (1881–1973), had their drawings and paintings inspired by personal dramas involving migraine aura. These artists likely incorporated their personal experiences with migraines, particularly visual aspects such as sparkling or flashing lights, and rings of fire, into their artworks. Their paintings often closely resemble the visual characteristics of migraine aura, revealing the potential intricate relationship between neurological phenomena and artistic inspiration. 15 16 17 18

The present article reevaluates Tarsila do Amaral's “Abaporu” through a neurological lens, proposing that its exaggerated proportions and chromatic intensity may evoke perceptual distortions commonly associated with migraine aura and AIWS. By bridging the domains of art and neuroscience, this study aims to uncover new dimensions in understanding how neurological conditions can illuminate aspects of Amaral's work, challenging conventional interpretations and inviting a fresh perspective on the intersection between perception and artistic creation.

## METHODS

We conducted a structured review of primary and secondary sources on Tarsila do Amaral's artistic production. This included systematic consultation of *Catálogo Raisonné Tarsila do Amaral* , Amaral's 1939 essay “Pintura Pau-Brasil e Antropofagia,” and the exhibition catalogue “Tarsila do Amaral: Inventing Modern Art in Brazil.” In addition, a systematic literature search was performed using PubMed and Google Scholar, focusing on Tarsila do Amaral's life and on neurological conditions relevant to the study, such as migraine aura and AIWS. No documentary evidence of migraine or aura was identified in the biographical sources. The analysis, therefore, concentrated on documenting the recurrent use of disproportion and gigantism across Amaral's production, and on assessing the plausibility of a neuroesthetic hypothesis in the context of her established art-historical program.

## RESULTS

**Figure 1:**
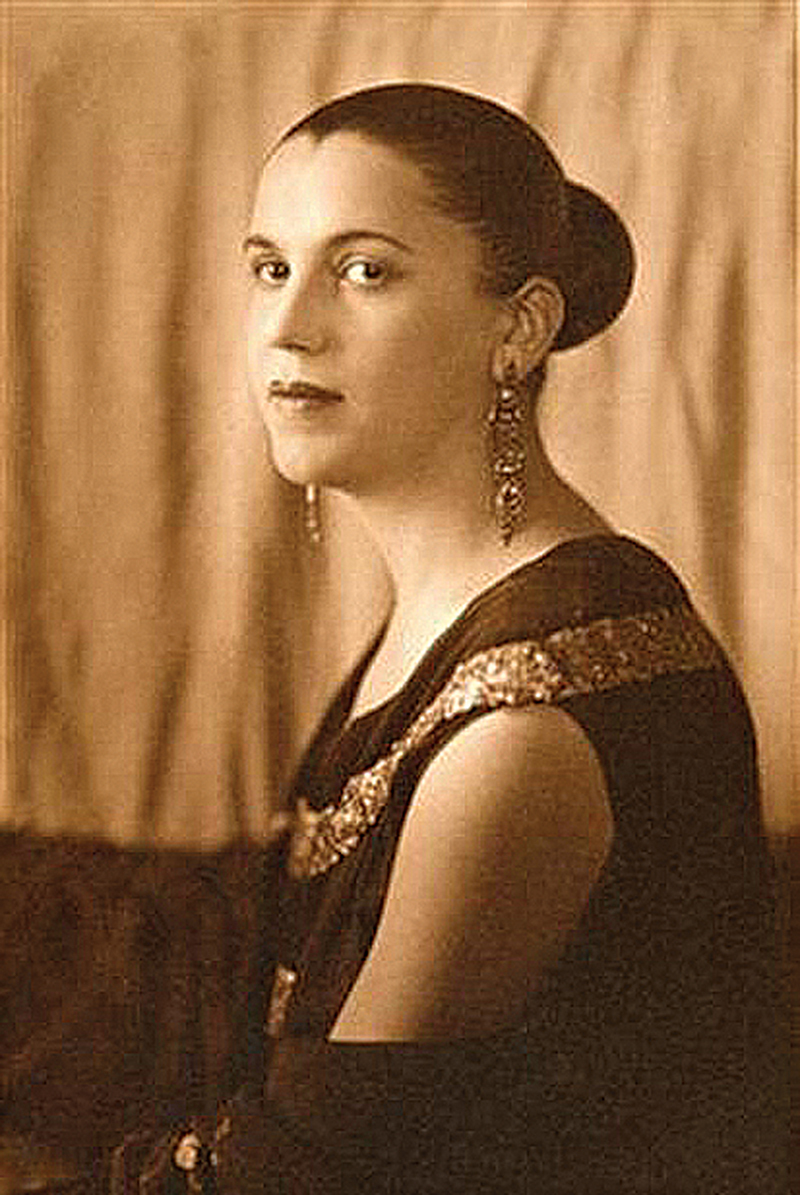
Source: Archives of Women Artists, Research and Exhibitions (AWARE). Tarsila do Amaral, author of “Abaporu”.

Tarsila do Amaral (1886–1973) is recognized as the greatest Brazilian painter of all time ( Figure 1 ). Born in Capivari, a small town in the countryside of the state of São Paulo, she had access to the arts from an early age. After completing her educational and artistic training in Spain and Paris, she returned to Brazil in June 1922, after a cultural effervescence resulting from the Modern Art Week, a major cultural event that took place in February 1922. The Modern Art Week sought the renewal of Brazilian art, until then strongly influenced by European art. 1 19

Although not present at that event, Tarsila soon became a leading exponent of modernist esthetics, developing a distinctive style marked by vivid color palettes and deliberate distortions of form and scale. Tarsila's artistic style was greatly influenced by cubism and surrealism and can be divided into 3 phases: Pau-Brasil (1924–1927), Anthropophagy (1928–1930), and the later Social phase (1930s onward). Among the countless paintings she produced, “A negra” (1923), “Abaporu” (1928), and “Operários” (1933) stand out. 1

Created in 1928 as a birthday gift to her then-husband, the writer Oswald de Andrade (1890–1954), “Abaporu” (from the Tupi-Guarani language, “man who eats human flesh”) depicts a seated figure of monumental proportions with oversized feet and a disproportionately small head. The painting soon became the most famous and influential work of Brazilian modernism and marked the beginning of the Anthropophagic phase, whose program sought to “devour” European culture—then dominant in Brazil—transforming it into something distinctly national. 20

However, systematic consultation of the *Catálogo Raisonné* and major exhibition catalogues indicates that the use of disproportion and gigantism was not limited to “Abaporu” but recurs throughout Amaral's production of the 1920s. Works such as “A negra” (1923) and “Antropofagia” (1929) reveal the same programmatic manipulation of scale, alternating monumental extremities with reduced cranial proportions. 21 22 These recurrent patterns demonstrate that “Abaporu” should be understood as part of a deliberate and sustained esthetic strategy of deformation, rather than as an isolated experiment.

## DISCUSSION

The present analysis situates “Abaporu” both within the larger trajectory of Tarsila do Amaral's artistic program and within the interpretive frame of neuroesthetics. Although previous scholarship often highlights the uniqueness of this painting in Brazilian modernism, its use of disproportion and gigantism is in fact consistent with strategies already explored in other works of the 1920s. Paintings such as “A negra” (1923) and “Antropofagia” (1929) display recurrent manipulations of scale, alternating monumental limbs with strikingly small heads. This continuity suggests that the visual distortions in “Abaporu” should not be regarded as an isolated invention but rather as part of Amaral's broader stylistic repertoire during the Pau-Brasil and Anthropophagic phases. 21 22

Scholars have frequently noted Amaral's deployment of gigantism—a calculated technique of monumental enlargement—as a defining feature of these periods. The hypertrophied feet and arms of “Abaporu”, as well as the monumental figure of “A negra”, exemplify this consistent approach. Far from reflecting pathology, such gigantism functioned as a metaphor for primitivism, fecundity, and cultural renewal, resonating with the Anthropophagic movement's call to “devour” and transform European traditions into a distinctly Brazilian modernism. This perspective underscores that the deformations addressed in our neurological reading were firmly embedded in Amaral's esthetic program, while still echoing perceptual phenomena familiar to clinical neurology. 22

**Figure 2:**
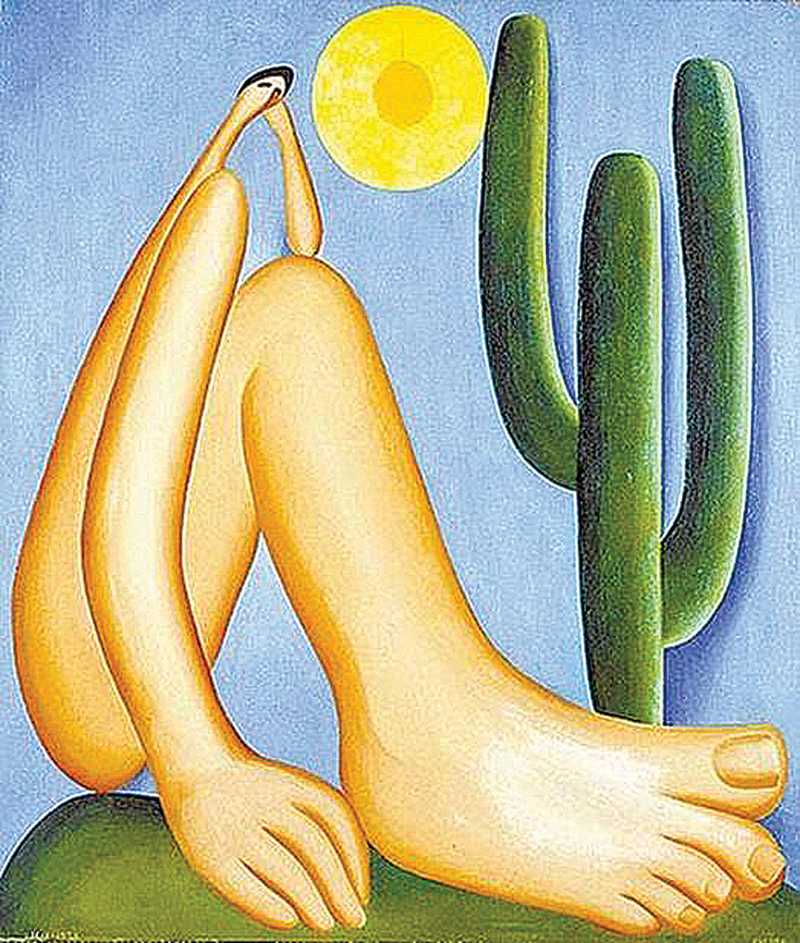
Source: Collection of Museo de Arte Latinoamericano de Buenos Aires (MALBA), Buenos Aires, Argentina. “Abaporu” (1928), by Tarsila do Amaral. Note the disproportion in the representation of the head, hand, and foot, referring to micropsia and macropsia, suggestive aspects of Alice in Wonderland Syndrome, common in migraine aura patients.

**Figure 3:**
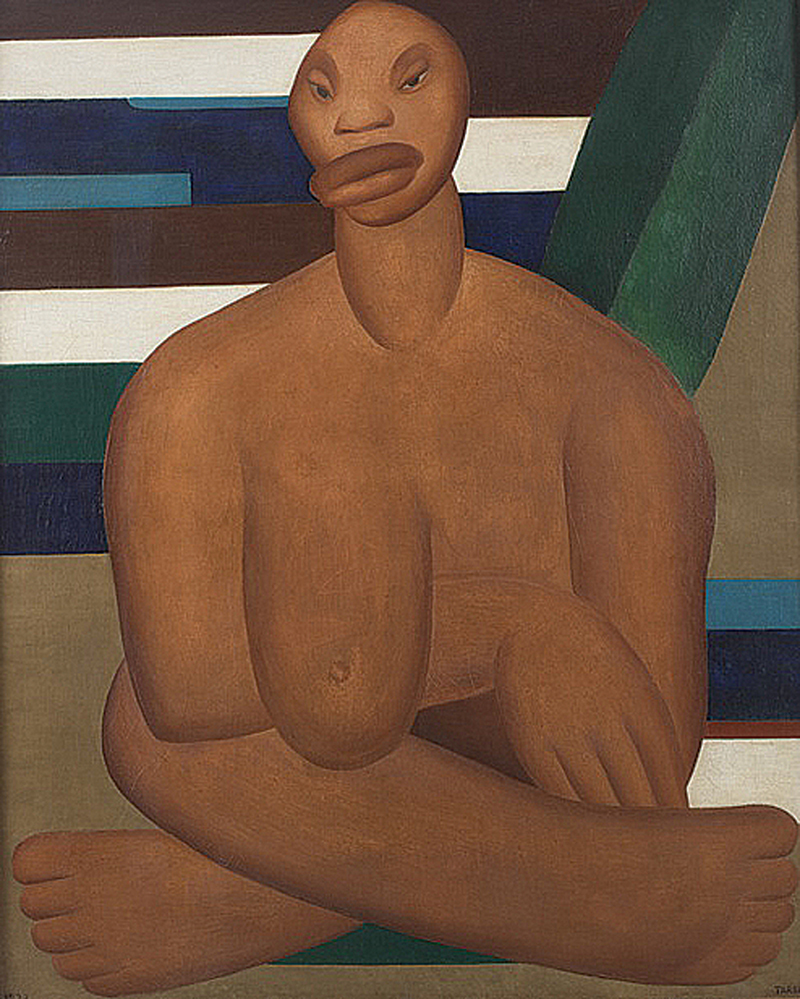
Source: Collection of Museu de Arte Contemporânea da Universidade de São Paulo (MAC USP), São Paulo, SP, Brazil. “A negra” (1923), by Tarsila do Amaral. The figure's monumental limbs and prominent lips contrast with the disproportionately small head, exemplifying the artist's technique of *gigantismo* .

Amaral herself reinforced this view in a 1939 essay, in which she described “Abaporu” as “a monstrous solitary figure, with immense feet, seated on a green plain, the arm bent resting on one knee, the hand sustaining the featherweight of the tiny head” ( Figure 2 ) explicitly linking the painting to the origins of the Anthropophagic movement. In that essay, Amaral also described “A negra” in strikingly similar terms, as “a seated figure with two robust masses of crossed legs, a heavy breast weighing upon the arm, enormous drooping lips, and a proportionally small head” ( Figure 3 ). 23 Such firsthand testimony makes it clear that distortion was not incidental, but central to her creative project. Anchoring the interpretation in the artist's own voice provides an important art-historical counterpoint to neuroesthetic readings.

In addition to the artist's own account, several art critics have emphasized the sociocultural dimension of “Abaporu”. The monstrous figure, with its enormous feet firmly planted on Brazilian soil next to a cactus, and its disproportionately small head contrasted with oversized hands, has been read as symbolizing the exaltation of physical and manual labor over intellectual pursuits, a commentary on the Brazilian society of the 1920s. 2 19 20 This interpretation underscores that Amaral's formal distortions were not only esthetic strategies, but also means for cultural critique, further enriching the multiple layers of meaning embedded in the work.

Within this art-historical frame, however, the neuroesthetic hypothesis becomes more compelling. The disproportionate forms in “Abaporu” evoke perceptual alterations reminiscent of macropsia and micropsia, phenomena associated with AIWS and often described by individuals experiencing migraine aura. In addition, the melancholic expression conveyed by the small, bowed head parallels the emotional burden of migraine aura, which is frequently accompanied by psychiatric comorbidities such as depression, generalized anxiety disorder, panic attacks, and phobias. 24 These conditions occur more often among people with migraine aura than in the general population and may be up to 10 times more common, substantially impairing quality of life. 25 26 27 From this perspective, the sadness implicit in the figure can be understood as mirroring the affective dimension of migraine.

The intense palette and bold outlines of “Abaporu” provide another layer to this interpretation, as the use of vibrant colors and dynamic contours may recall the heightened sensory sensitivity and perceptual overload often reported by individuals with migraine. In this sense, the painting may be read as a visual analogue of the perceptual distortions, mood shifts, and sensory disturbances characteristic of migraine aura. Importantly, this reading does not seek to assign a retrospective medical diagnosis to Amaral, but rather highlights how artistic intuition can converge with phenomena later described in neurological literature. Viewed from this perspective, “Abaporu” emerges as a work that invites readers to explore the intersection of art and neuroscience, challenging conventional notions of perception and reality.

Biographical elements of Amaral's life further contextualize these possibilities. Accounts of marital problems, family losses, and her paraplegia following complications of spinal surgery describe periods of profound sadness and depression. 1 Yet no historical evidence documents migraine episodes. This silence does not completely rule out the hypothesis, since Amaral might have experienced aura without headache, a condition often overlooked. Moreover, because AIWS was only described in 1955—decades after “Abaporu” was painted—any such experiences would likely have gone unrecognized or concealed, consistent with the stigma surrounding unexplained neurological symptoms at the time. 19

In conclusion, the interpretation herein presented places “Abaporu” simultaneously within Amaral's programmatic deformation of scale and within a neuroesthetic view. These perspectives are not mutually exclusive: the former explains the artist's deliberate esthetic strategies, while the latter enriches understanding of how such visual devices resonate with neurological models of altered perception. Together, they underscore the potential of interdisciplinary dialogue to expand both art-historical and neuroscientific horizons.

Speculating on the relationship between neurological conditions and works of art is inevitably challenging. Nevertheless, history shows that many artists have consciously or unconsciously drawn on their physical or psychological experiences as sources of creative inspiration. Revisiting the conception of “Abaporu,” our analysis does not claim that Tarsila do Amaral suffered from migraine with aura or AIWS. Rather, it highlights how the painting's distortions of scale, chromatic intensity, and affective charge resonate with perceptual and emotional phenomena later described in neurology. Recognizing this convergence does not diminish Amaral's originality; instead, it enriches our understanding of her artistic strategies, situating them at the crossroads of modernist experimentation, cultural critique, and the embodied experience of perception. This perspective underscores the value of interdisciplinary dialogue in broadening both art-historical and neuroscientific horizons.
